# An Al-doped SrTiO_3_ photocatalyst maintaining sunlight-driven overall water splitting activity for over 1000 h of constant illumination†
†Electronic supplementary information (ESI) available: Time course of the water splitting activity, XPS spectra, and experimental setup for operando XAFS spectroscopy. See DOI: 10.1039/c8sc05757e


**DOI:** 10.1039/c8sc05757e

**Published:** 2019-01-24

**Authors:** Hao Lyu, Takashi Hisatomi, Yosuke Goto, Masaaki Yoshida, Tomohiro Higashi, Masao Katayama, Tsuyoshi Takata, Tsutomu Minegishi, Hiroshi Nishiyama, Taro Yamada, Yoshihisa Sakata, Kiyotaka Asakura, Kazunari Domen

**Affiliations:** a Department of Chemical System Engineering , School of Engineering , The University of Tokyo , 7-3-1 Hongo , Bunkyo-ku , Tokyo 113-8656 , Japan . Email: domen@chemsys.t.u-tokyo.ac.jp; b Center for Energy & Environmental Science , Interdisciplinary Cluster for Cutting Edge Research , Shinshu University , 4-17-1 Wakasato , Nagano-shi , Nagano 380-8553 , Japan; c Graduate School of Sciences and Technology for Innovation , Yamaguchi University , 2-16-1 Tokiwadai , Ube-shi , Yamaguchi 755-8611 , Japan; d Blue Energy Center for SGE Technology , Yamaguchi University , 2-16-1 Tokiwadai , Ube-shi , Yamaguchi 755-8611 , Japan; e Institute for Catalysis , Hokkaido University , Kita 21 Nishi 10, Kita-ku , Sapporo-shi , Hokkaido 001-0021 , Japan

## Abstract

Photocatalytic water splitting is a viable approach to the large-scale production of renewable solar hydrogen. The apparent quantum yield for this reaction has been improved, but the lifespan of photocatalysts functioning under sunlight at ambient pressure have rarely been examined, despite the critical importance of this factor in practical applications. Herein, we show that Al-doped SrTiO_3_ (SrTiO_3_:Al) loaded with a RhCrO_*x*_ (rhodium chromium oxide) cocatalyst splits water with an apparent quantum yield greater than 50% at 365 nm. Moreover, following the photodeposition of CoOOH and TiO_2_, this material maintains 80% of its initial activity and a solar-to-hydrogen energy conversion efficiency greater than or equal to 0.3% over a span of 1300 h under constant illumination by simulated sunlight at ambient pressure. This result is attributed to reduced dissolution of Cr in the cocatalyst following the oxidative photodeposition of CoOOH. The photodeposition of TiO_2_ further improves the durability of this photocatalyst. This work demonstrates a concept that could allow the design of long-term, large-scale photocatalyst systems for practical sunlight-driven water splitting.

## Abstract 2

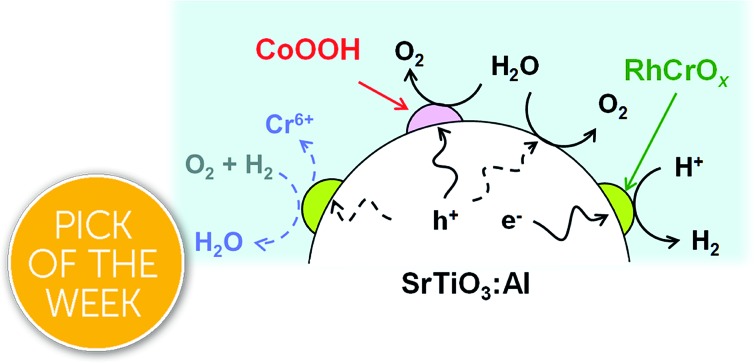
The development of robust and efficient water splitting photocatalysts overcomes a long-standing barrier to sustainable large-scale solar hydrogen evolution systems.

## Introduction

The development of renewable energy resources is an important issue, owing to increasing concerns regarding environmental degradation and limited fossil fuel supplies. Photocatalytic overall water splitting has attracted much attention for the past several decades owing to its potential for the large scale production of hydrogen as a renewable fuel.^1^–^4^ Certain metal oxide photocatalysts have shown apparent quantum yields (AQYs) greater than 50% during overall water splitting in response to irradiation with UV light.^5^–^7^ Photocatalytic systems exhibiting relatively high AQYs under visible light have also recently emerged.^8^–^10^ However, the practical application of photocatalytic water splitting requires systems that provide both a high level of activity and durability over prolonged operation periods.^11^ La-doped NaTaO_3_ loaded with NiO has been reported to maintain its water splitting activity over a span of 16 days without noticeable degradation at ambient pressure under UV illumination.^5^ A solid solution of GaN and ZnO (GaN:ZnO) loaded with rhodium chromium oxide (RhCrO_*x*_) as a hydrogen evolution cocatalyst also demonstrated constant water splitting activity over a span of three months under visible light at a reduced pressure, although this material underwent a 50% loss in activity over the following three months.^12^ This activity loss was attributed to oxidative dissolution of the Cr component of the RhCrO_*x*_ (which is essential for suppressing backward reactions), and it was found that such dissolution could be reduced by coloading RuO_*x*_ as an oxygen evolution cocatalyst. Interestingly, the durability of photocatalytic systems during overall water splitting under sunlight at ambient pressure has rarely been investigated, even though these are the conditions that would be present during the practical application of such systems.

The present work examined the long-term durability of RhCrO_*x*_-loaded SrTiO_3_:Al (RhCrO_*x*_/SrTiO_3_:Al). This material was selected for study because it is one of the most active photocatalysts for overall water splitting under sunlight and has a high AQY in the near UV region (56% at 365 nm).^7^ The results presented herein demonstrate that the oxidative photodeposition of CoOOH on the photocatalyst stabilises the Cr in the RhCrO_*x*_ cocatalyst, which would otherwise be oxidised and dissolved. The concurrent deposition of TiO_2_ further improves the durability and allows this photocatalyst, once fixed on a substrate, to maintain 80% of its initial activity over 1300 h of constant simulated sunlight illumination at ambient pressure.

## Results and discussion

SrTiO_3_:Al was prepared using a flux method and the RhCrO_*x*_ cocatalyst was loaded by impregnation followed by calcination in air.^7^ Cobalt species were loaded onto the resulting RhCrO_x_/SrTiO_3_:Al by photodeposition.^7^ Amorphous TiO_2_ was also loaded on some samples of this material by photodeposition.^13^ Water splitting reactions were carried out using a gas flow reaction system or a panel reactor. A 300 W Xe lamp (300 nm < *λ* < 500 nm), a 450 W high-pressure Hg lamp (*λ* > 300 nm) and a solar simulator (100 mW cm^–2^, AM 1.5G) were used as light sources for standard, accelerated deactivation and long-term durability tests, respectively.

**Fig. 1:**
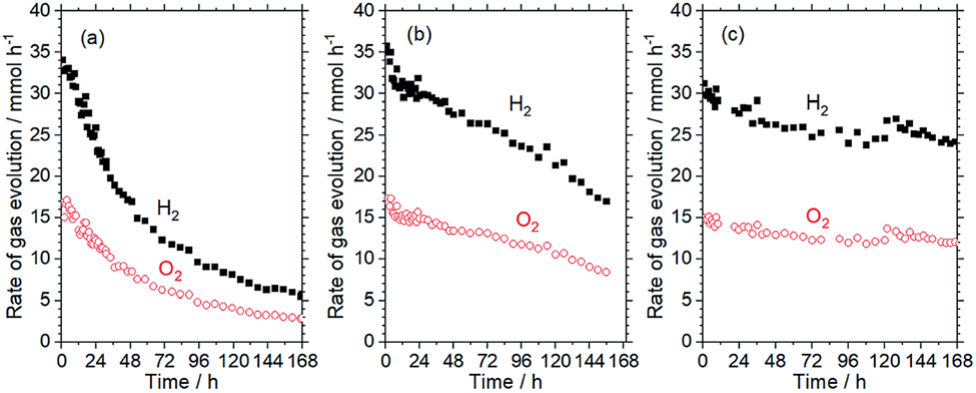
Time courses of water-splitting activity of SrTiO_3_:Al loaded with (a) RhCrO_*x*_, (b) RhCrO_*x*_ and 0.3 wt% Co species, and (c) RhCrO_*x*_, 0.3 wt% Co species and 3 wt% TiO_2_. Reaction conditions: photocatalyst, 0.5 g; reaction solution, 370 mL distilled water; reaction temperature, 291 K; light source, 450 W high-pressure Hg lamp (*λ* > 300 nm).

Fig. 1a shows the time course of the water splitting activity of RhCrO_*x*_/SrTiO_3_:Al in response to irradiation by the high-pressure Hg lamp, and demonstrates that the water splitting rate was decreased to 14% of its original value after 168 h. Inductively-coupled plasma optical emission spectroscopy (ICP-OES) analyses determined that 37% of the Cr in the RhCrO_*x*_ cocatalyst was dissolved following this trial, although there was no significant leaching of any other elements. This result is in agreement with the dissolution of Cr species that has been reported for RhCrO_*x*_/GaN:ZnO.^12^ The Cr in RhCrO_*x*_ is thought to prevent backward reactions involving molecular oxygen,^14^ similar to the case for core/shell Rh/Cr_2_O_3_ cocatalysts.^15^ Thus, loss of the Cr species would be expected to result in exposure of the Rh species and an increase in the extent of backward reactions. Additional trials showed that the degree of deactivation was reduced under weaker irradiation when the same amounts of water were decomposed (see Fig. S1 in ESI†). These data suggest that, if the photoexcited holes generated by irradiation are not used promptly, they may oxidise Cr^3+^ in the RhCrO_*x*_ cocatalyst to soluble Cr^6+^ species.

Cobalt oxide (CoO_*y*_) is known to extract holes from photocatalysts and to simultaneously function as an oxygen evolution cocatalyst.^16^ On this basis, cobalt species were loaded *via* photodeposition using CoCl_2_ in the present work. The aim was to suppress the oxidative corrosion of Cr^3+^ species by excess holes and thus prevent the deactivation of the RhCrO_*x*_/SrTiO_3_:Al photocatalyst. As shown in Fig. S2,† the RhCrO_*x*_/SrTiO_3_:Al maintained only 34% of its initial activity after 72 h under UV irradiation by the Xe lamp. Following the addition of Co species, an induction period was observed, during which the water splitting rate gradually increased. Notably, RhCrO_*x*_/SrTiO_3_:Al loaded with 0.3 wt% Co maintained stable activity under these conditions. When the Co loading was increased to 0.5 wt%, the induction period was prolonged while the water splitting rate stabilized at a lower value, suggesting excessive loading of the Co species. The water splitting reaction was also carried out using RhCrO_*x*_/SrTiO_3_:Al coloaded with 0.3 wt% Co species in conjunction with irradiation by the high-pressure Hg lamp (Fig. 1b). The results show that the durability of the photocatalyst was improved without sacrificing activity by loading an appropriate amount of Co species.

Fig. S3† and Table 1 provide Cr and Ti 2p X-ray photoelectron spectroscopy (XPS) data and the surface Cr/Ti atomic ratios for RhCrO_*x*_/SrTiO_3_:Al with and without the coloading of 0.3 wt% Co species, and before and after the water splitting reaction was conducted for a span of one week. In the absence of Co loading, the Cr/Ti ratio decreased from 0.12 to 0.07 during the reaction. Adding the Co species increased the Cr/Ti ratio from 0.12 to 0.21, suggesting that the Co species was photodeposited on the SrTiO_3_:Al photocatalyst rather than on the RhCrO_*x*_ cocatalyst. Notably, the presence of the Co species decreased the reduction in the Cr/Ti ratio during the reaction, such that the ratio was reduced from 0.21 only to 0.20. Moreover, the concentration of Cr ions in the reaction solution was below the level in a blank solution (7.3 ppb, as determined by ICP-OES), indicating negligible Cr dissolution. The Co/Ti ratio was also not decreased significantly during the reaction, demonstrating the robust nature of the Co species in this material. Note that the activity of CoOOH/RhCrO_*x*_/SrTiO_3_:Al decreased to approximately the half although the loss of Cr content on the RhCrO_*x*_ cocatalyst was marginal. The activity in overall water splitting is governed by many factors and the degree of deactivation would not be linear to the respective factors, including the loss of Cr species. Nevertheless, this observation implies that factors other than the loss of Cr species may be identified as additional causes of the deactivation.

**Table 1:** Surface atomic ratios of cocatalyst-loaded SrTiO_3_:Al before and after water splitting reactions under irradiation by a high-pressure Hg lamp (*λ* > 300 nm)

Cocatalyst	Reaction time/h	Cr/Ti	Co/Ti
RhCrO_*x*_	1	0.12	—
	168	0.07	—
CoOOH/RhCrO_*x*_	0	0.21	0.27
	168	0.20	0.25
TiO_2_/CoOOH/RhCrO_*x*_	0	0.06	0.04
	168	0.06	0.04

**Fig. 2:**
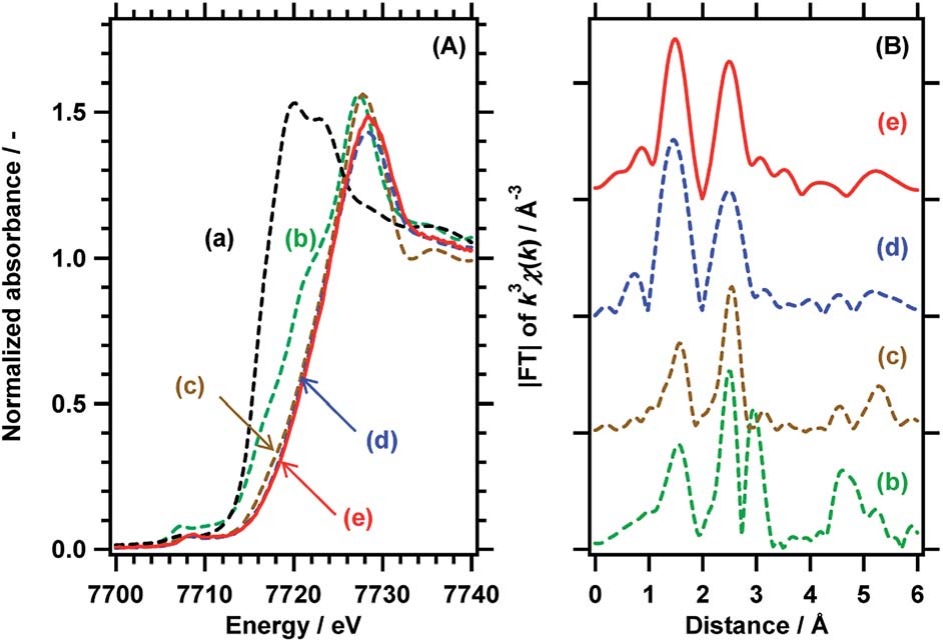
(A) Co-K edge XANES data and (B) Fourier transforms of *k*^3^-weighted Co-K EXAFS spectra for (a) CoCl_2_·6H_2_O, (b) Co_3_O_4_, (c) CoOOH, and RhCrO_*x*_/SrTiO_3_:Al coloaded with 0.3 wt% Co species (d) in darkness and (e) following UV illumination for 15 h by a 300 W Xe lamp.

The local structure and the valence state of the Co species loaded at the optimum concentration (0.3 wt%) were investigated by X-ray absorption fine structure (XAFS) analysis in distilled water under UV illumination as well under dark conditions. Fig. 2 shows Co-K edge X-ray absorption near edge structure (XANES) data and the Fourier transformed *k*^3^-weighted extended XAFS (EXAFS) spectra for RhCrO_*x*_/SrTiO_3_:Al loaded with 0.3 wt% Co species. The photodeposited Co species exhibit an absorption edge at an energy greater than the value for the CoCl_2_·6H_2_O used as a precursor, and the present specimen generated a XANES spectrum similar to that of CoOOH.^17^,^18^ This result indicates that CoOOH was photodeposited on the oxidation sites (*i.e.*, the bare surface of the SrTiO_3_:Al). The chemical state of the deposited Co species was also confirmed from the local structure data. The valence state and structure of the photodeposited CoOOH were unchanged during 15 h of UV illumination, in contrast to the behaviour of Co-based electrocatalysts, in which Co^2+^ is oxidized to Co^3+^ and Co^4+^, after which the latter species oxidise OH^–^ to O_2_.^19^–^21^ The Co in the present work likely maintained a constant valence state because the quasi-Fermi level for holes in the photocatalyst was determined by the balance between the hole concentration and charge generation/consumption (by surface reactions or recombination). Conversely, the potential for electrocatalysts can be externally fixed at specific values. Additionally, the XAFS data for the Rh species in the RhCrO_*x*_ cocatalyst in aqueous solution, both under darkness and following the water splitting reaction, closely matched the results expected for trivalent Rh in a corundum structure. This structure is also essentially equivalent to that of RhCrO_*x*_ loaded on GaN:ZnO.^22^ Therefore, the bulk chemical states of the CoOOH and RhCrO_*x*_ cocatalysts were both unchanged during the photocatalytic water splitting reaction.

**Fig. 3:**
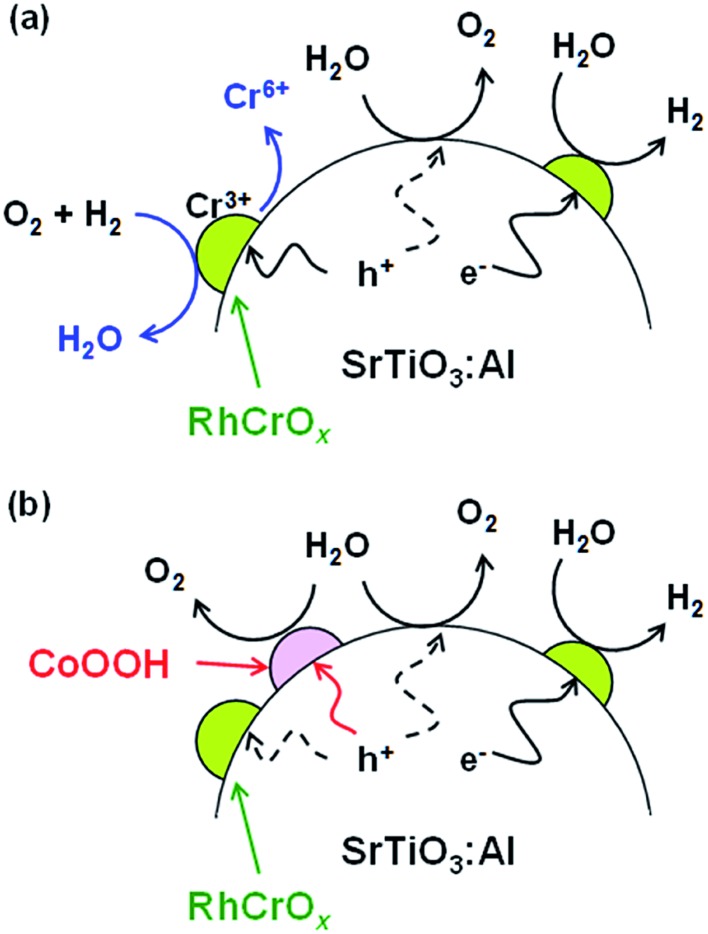
Illustrations of (a) RhCrO_*x*_/SrTiO_3_:Al deactivation mechanism and (b) stabilisation of RhCrO_*x*_/SrTiO_3_:Al by CoOOH. The stoichiometry of the reactions is ignored for simplicity.

The function of CoOOH present on the RhCrO_*x*_/SrTiO_3_:Al surface can be interpreted as follows. In the absence of CoOOH (Fig. 3a), photoexcited holes oxidise not only water but also the RhCrO_*x*_, leading to the dissolution of Cr.^12^ As a result, backward reactions occur on the Cr-deficient RhCrO_*x*_ cocatalyst exposed to the reaction solution. CoOOH loaded on the oxidation sites is believed to collect photoexcited holes (Fig. 3b). Thus, because holes are more likely to participate in the water oxidation reaction as opposed to reacting with the RhCrO_*x*_, oxidative corrosion of the RhCrO_*x*_ cocatalyst and thereby the deactivation of the photocatalyst is suppressed. However, it is uncertain whether water oxidation occurs exclusively on CoOOH because RhCrO_*x*_/SrTiO_3_:Al exhibited the similar initial activities before and after the coloading of CoOOH (Fig. 1a and b). Effects of CoOOH coloading on the carrier dynamics and surface kinetics may be analysed by transient absorption spectroscopy in the presence of reactants.^23^

**Fig. 4:**
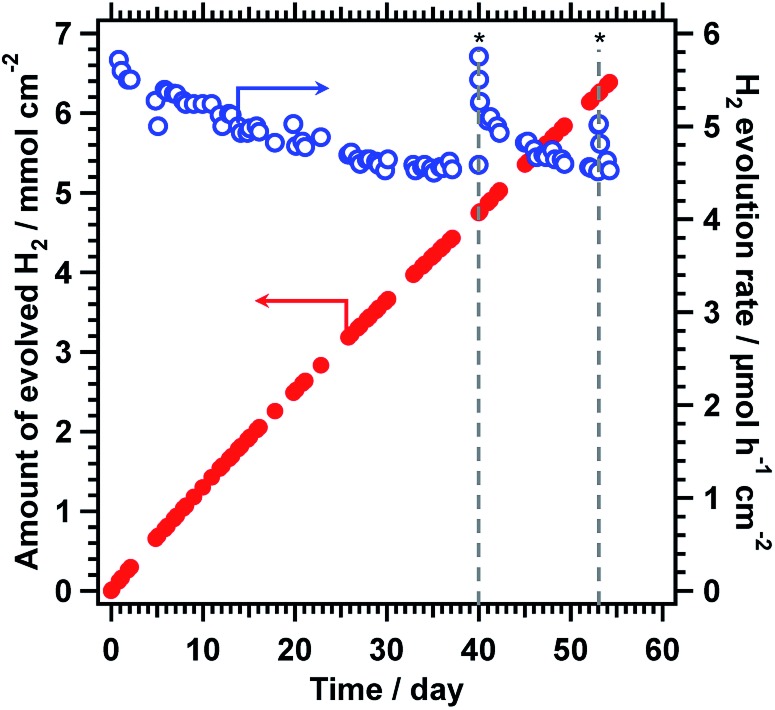
Time course of water splitting activity of 5 × 5 cm^2^ TiO_2_/CoOOH/RhCrO_*x*_/SrTiO_3_:Al sheet in panel reactor. Reaction conditions: photocatalyst, 20 mg; reaction solution, 1 mm deep distilled water; reaction temperature, 291 K; light source, AM 1.5G solar simulator. Asterisk symbols (*) indicate interruption of the illumination for two days.

The durability of the CoOOH/RhCrO_*x*_/SrTiO_3_:Al photocatalyst was further enhanced by the photodeposition of amorphous TiO_2_ (3 wt%), as shown in Fig. 1c. This TiO_2_/CoOOH/RhCrO_*x*_/SrTiO_3_:Al photocatalyst was processed into 5 × 5 cm sheets and the durability of the material was examined in a panel-type reactor with a water depth of 1 mm under simulated sunlight at ambient pressure, to simulate a realistic operational mode.^7^ These sheets initially exhibited a water splitting rate of 5.7 μmol h^–1^ cm^–2^ and a solar-to-hydrogen conversion efficiency (STH) of 0.4% during the early stage of illumination. The activity gradually decreased by 20% over 1300 h of constant illumination (8.6 kJ cm^–2^ day^–1^) but the catalyst sheet maintained a minimum 0.3% STH throughout (Fig. 4). This time span corresponds to 173 days assuming an insolation of 2.7 kJ cm^–2^ day^–1^.^11^ Notably, the photocatalyst sheet recovered its original water splitting activity after being kept in darkness and maintained this recovered activity for several days of additional illumination. These characteristics would be advantageous during operation under natural sunlight. In comparison, RhCrO_*x*_/SrTiO_3_:Al modified only with CoOOH exhibited an initial water splitting rate of 5.0 μmol h^–1^ cm^–2^ and lost 40% of its original activity after 40 days under the identical reaction conditions.^7^ Therefore, the photodeposition of TiO_2_ evidently enhanced the durability of the CoOOH/RhCrO_*x*_/SrTiO_3_:Al sheet while maintaining a high STH during overall water splitting. In comparison, direct deposition of TiO_2_ onto RhCrO_*x*_/SrTiO_3_:Al lowered the water splitting activity while enhanced the durability (Fig. S4 in ESI†). Amorphous TiO_2_ has been reported to form an insoluble, selectively permeable layer covering the entire surface of the cocatalyst/photocatalyst composite and preventing backward reactions.^13^ The XPS data presenting the decrease in both the Cr/Ti and Co/Ti ratios support this scenario (Table 1). It is believed that the TiO_2_ played similar roles in the present work, and may also have suppressed detachment of the cocatalysts by the motion of the reaction media. Excessively thick TiO_2_ coating would induce mass transfer resistances and lower the water splitting activity. However, it is difficult to precisely characterise the function and structure of the TiO_2_ layers in this work, because Ti is also a main component of the photocatalyst itself. Our group intends to perform a more detailed analysis of the degradation behaviour in future, once the appropriate experimental protocols are established.

## Conclusions

A RhCrO_*x*_/SrTiO_3_:Al photocatalyst highly active during overall water splitting was found to be stabilised *via* the photodeposition of CoOOH from CoCl_2_. The presence of CoOOH suppressed the oxidative loss of the Cr component of the RhCrO_*x*_ cocatalyst. The photocatalyst could be further stabilised by photodeposition of TiO_2_, such that it maintained 80% of its initial activity during 1300 h of constant AM 1.5G irradiation at ambient pressure. This same material exhibited an STH of 0.3% or greater throughout the duration of the test when in the form of a photocatalyst sheet contained in a panel-type reactor. The durability of such sheets under natural sunlight could be further extended as the result of an apparent healing effect under dark conditions. The development of robust water splitting photocatalysts, as demonstrated herein, overcomes a long-standing barrier to practical applications. This technique is expected to assist in the development of sustainable large-scale solar hydrogen evolution systems.

## Experimental section

SrTiO_3_:Al was prepared using a flux method previously reported by our group.^7^ In this process, SrTiO_3_ (99.9%, Wako), Al_2_O_3_ nanopowder (Aldrich) and SrCl_2_ (98.0%, Kanto) were mixed at a molar ratio of 1 : 0.02 : 10 using an agate mortar. The mixture was then heated in an alumina crucible at 1423 K for 10 h and subsequently cooled to room temperature, after which the product was stirred in a large volume of distilled water and then retrieved by filtration to remove impurities associated with the SrCl_2_ flux. This rinsing process was repeated three times, after which the resulting SrTiO_3_:Al powder was dried at 313 K in an oven.

This SrTiO_3_:Al powder was loaded with RhCrO_*x*_ (0.1 wt% Rh and 0.1 wt% Cr) using an impregnation method,^7^ employing Na_3_RhCl_6_·*n*H_2_O (17.8 wt% Rh, Mitsuwa) and Cr(NO_3_)_3_·9H_2_O (98.0–103.0%, Kanto) as the Rh and Cr sources, respectively. In this process, SrTiO_3_:Al powder was dispersed in an aqueous solution containing the required amounts of the Rh and Cr precursors, and sonicated for several minutes. Following this, the solution was evaporated to dryness while being stirred, and the resulting solid was calcined in air at 623 K for 1 h. The AQY of the resulting RhCrO_*x*_/SrTiO_3_:Al during overall water splitting was determined to be 54% at 365 nm, which was consistent with our previous work.^7^

A gas flow system was used for photocatalytic water splitting reactions. The system was first purged with a mixture of gaseous Ar and N_2_ at ambient pressure, using the N_2_ as an internal standard for quantification purposes. A photocatalyst sample (0.1 g) was suspended in distilled water (100 mL) in a top-irradiation reactor, and irradiated through a Pyrex window using a 300 W xenon lamp (300 nm < *λ* < 500 nm) equipped with a dichroic mirror. In some experiments, a 50% transmission neutral density filter was inserted to reduce the light intensity. Trials were also performed with irradiation by a 450 W high-pressure Hg lamp (*λ* > 300 nm) to accelerate the water splitting reaction and thereby promote the deactivation of the photocatalyst, using an inner irradiation cell made of Pyrex glass containing a photocatalyst sample (0.5 g) suspended in distilled water (370 mL).

CoOOH was loaded onto RhCrO_*x*_/SrTiO_3_:Al by photodeposition at ambient pressure. The RhCrO_*x*_/SrTiO_3_:Al photocatalyst (0.5 g) was dispersed in distilled water (370 mL) containing CoCl_2_ and irradiated by a 450 W high-pressure Hg lamp (*λ* > 300 nm). Photodeposition was completed within 6 h. It was also found to be possible to deposit CoOOH using a top-irradiation cell under xenon lamp irradiation (see Fig. S2†), but a longer time span was required and the durability enhancement was less effective.

In preparation for the photodeposition of an amorphous TiO_2_ layer,^13^ titanium tetraisopropoxide (53 mg, 97.0%, Kanto) was added to 1 mL of an aqueous H_2_O_2_ solution (30%, Wako) and sonicated repeatedly for several minutes at a time until a pale yellow solution was obtained. An aqueous NaOH solution (1 M) was subsequently added to obtain a Na:Ti molar ratio of 2 : 1, which generated a transparent, light yellow solution. This solution was subsequently added to a reactant solution containing 0.5 g CoOOH/RhCrO_*x*_/SrTiO_3_:Al in an inner-irradiation cell so that the TiO_2_ concentration was 3 wt% with respect to the photocatalyst. The suspension was illuminated by a high-pressure Hg lamp (*λ* > 300 nm) at ambient pressure for at least 2 h. It was also possible to deposit TiO_2_ using a top-irradiation cell under xenon lamp irradiation. However, the durability enhancement was less effective when the resultant photocatalyst was processed into the form of sheets.

Samples were characterised by X-ray photoelectron spectroscopy (XPS; JPS-9000, JEOL) and X-ray absorption fine structure (XAFS) spectroscopy. The binding energies acquired by XPS were corrected using the Au 4f_7/2_ peak (84.0 eV) as a reference for each sample. Co-K edge XAFS data were acquired using the BL5S1 beamline at the Aichi Synchrotron Radiation Centre (Aichi, Japan), employing the CoOOH/RhCrO_*x*_/SrTiO_3_:Al photocatalyst (0.1 wt% Rh, 0.1 wt% Cr and 0.3 wt% Co). Fluorescence X-rays were detected using Si drift detectors. Rh-K edge XAFS data were obtained on the NW10A beamline (proposal no. 2017G076) at the Photon Factory of the High Energy Accelerator Research Organization (Tsukuba, Japan) using the CoOOH/RhCrO_*x*_/SrTiO_3_:Al photocatalyst (0.3 wt% Rh, 0.3 wt% Cr and 0.3 wt% Co). Fluorescence X-rays were detected with Ge solid-state detectors after passing through a Ru filter. XAFS analyses of the CoOOH/RhCrO_*x*_/SrTiO_3_:Al photocatalysts were performed using the operando technique in distilled water under UV irradiation as well as under dark conditions. The operando XAFS spectroscopy setup is pictured in Fig. S5.† CoOOH/RhCrO_*x*_/SrTiO_3_:Al (0.4 g) was suspended in distilled water (approximately 2 mL) in a Pyrex tube (inner diameter of 9 mm and outer diameter of 12 mm) that had a curved Kapton window along its lateral face, and was vigorously stirred using a magnetic stirrer. The photocatalyst suspension was irradiated with UV light through the glass tube, using a 300 W xenon lamp (300 nm < *λ* < 500 nm) equipped with a quartz optical fibre. Note that vigorous stirring was necessary because otherwise the photocatalyst powder was pushed upwards by bubbles of hydrogen and oxygen. Incident X-rays were targeted such that they irradiated the photocatalyst suspension by passing through the curved Kapton window but not through the glass tube. Fluorescence X-rays were also collected through the window. During the illumination process, the photocatalyst suspension was cooled with a fan. Reference samples were analysed in transmittance mode using pellets made of mixtures of commercial compounds and boron nitride, when available. Rh_0.5_Cr_1.5_O_3_ was prepared by a polymerised complex method.^22^ CoOOH was prepared by calcining commercial Co(OH)_2_ (97.0%, Kishida) in air.^16^ Reaction solutions were analysed by inductively-coupled plasma optical emission spectrometry (ICP-OES; ICPS-8100, Shimadzu).

Reactors of our own design were employed to assess the activity of TiO_2_/CoOOH/RhCrO_*x*_/SrTiO_3_:Al photocatalyst sheets. The RhCrO_*x*_/SrTiO_3_:Al photocatalyst powder was modified with CoOOH (0.3 wt% as Co) and TiO_2_ (3 wt% as TiO_2_) by photodeposition under Hg lamp irradiation for 6 and 3 h, respectively, prior to processing into sheets. The details regarding the processing of the photocatalyst into sheets and the design of the reactor have been described elsewhere.^7^ Distilled water was used as the reaction solution and the panel reactors were tilted at an angle of 20° (so that water bubbles could move upward as a result of the buoyant force) and were illuminated with a solar simulator (XES-40S2-CE, SAN-EI Electric) generating AM 1.5G irradiation (100 mW cm^–2^). The evolved gases were collected based on the upward displacement of water. The amount of hydrogen evolved was subsequently calculated based on the assumption that the molar ratio of the evolved hydrogen and oxygen was the expected stoichiometric value of 2 : 1.

## Conflicts of interest

There are no conflicts to declare.

## Author contributions

K. D. led the research project. T. Hisatomi and K. D. supervised the experimental work. H. L. prepared and analysed the photocatalyst and conducted the water splitting reactions. T. Hisatomi, Y. G., M. Y., T. Higashi, M. K. and K. A conducted the XAFS analyses. T. T. conceived the TiO_2_ coating and conducted the water splitting reactions using photocatalyst sheets. H. N. prepared the panel-type reactors. T. Y. performed ICP-OES analyses. H. L., T. Hisatomi, Y. G., M. Y., T. Higashi, M. K., T. T., T. M, Y. S., K. A. and K. D. discussed the results. H. L., T. H. and K. D. wrote the manuscript with contributions from the other authors. All authors reviewed the manuscript.

## Supplementary Material

Supplementary information

Video abstract
