# Supplementary material for: An Al-doped SrTiO3 photocatalyst maintaining sunlight-driven overall water splitting activity for over 1000 h of constant illumination
Source: Chem Sci. 2019 Jan 24;10(11):3196–201. doi: 10.1039/c8sc05757e (PMC6430014; doi:10.1039/c8sc05757e)
Supplement: Supplementary file 1 [file SC-010-C8SC05757E-s001.pdf]

This journal is © The Royal Society of Chemistry 2019

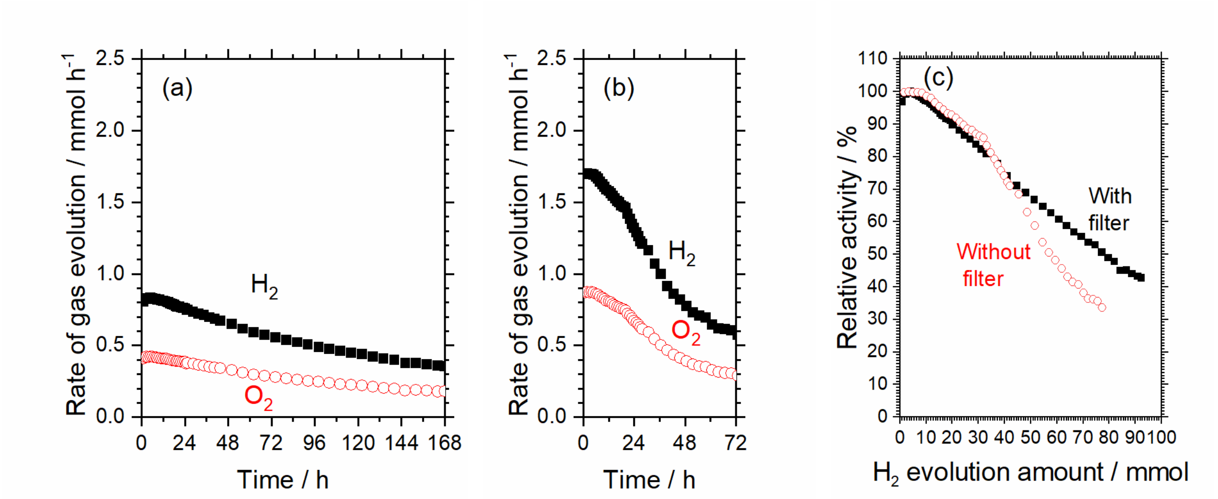

Figure S1. Time courses of water-splitting activity of $SrTiO_3$:Al loaded with $RhCrO_x$ (a) with and (b) without 50% transmission neutral density filter. (c) Relative activities as functions of H_2 evolution amount with and without filter in place. Reaction conditions: photocatalyst, 0.1 g; reaction solution, 100 mL distilled water; reaction temperature, 291 K; light source, 300 W Xe lamp (300 nm < λ < 500 nm).

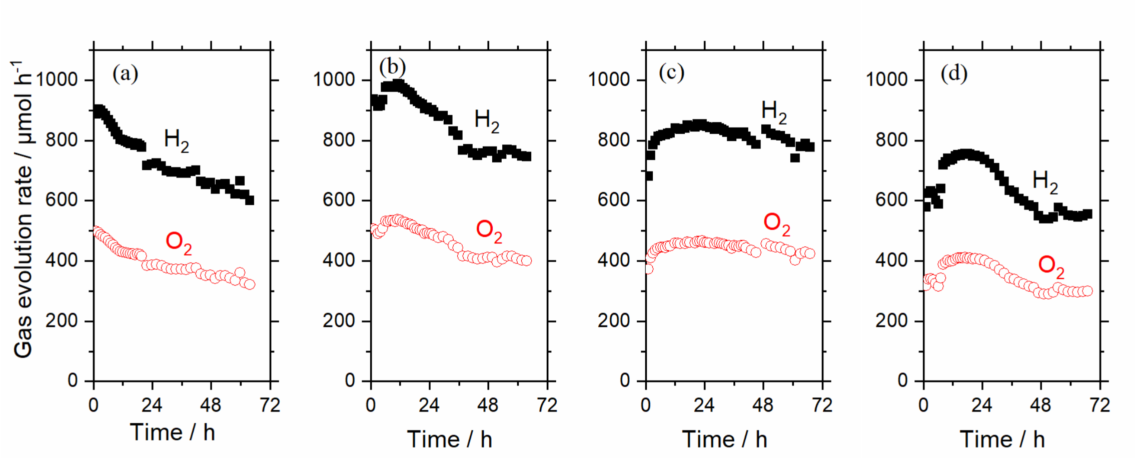

Figure S2. Time courses of water splitting activity of $RhCrO_x$/$SrTiO_3$:Al coloaded with Co species by *in situ* photodeposition at Co concentrations of (a) 0, (b) 0.1, (c) 0.3 and (d) 0.5 wt%. Reaction conditions: photocatalyst, 0.1 g; reaction solution, 100 mL distilled water; reaction temperature, 291 K; light source, 300 W Xe lamp (300 nm < λ < 500 nm).

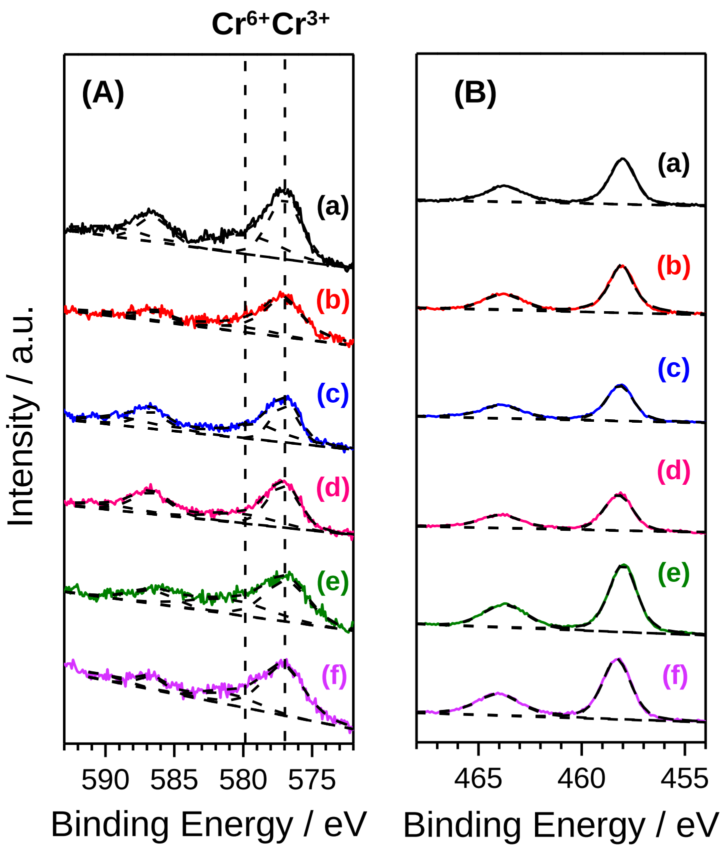

Figure S3. (A) Cr 2*p* and (B) Ti 2*p* XPS spectra for (a,b) $RhCrO_x/SrTiO_3$:Al, (c,d) $RhCrO_x/SrTiO_3$:Al coloaded with 0.3 wt% Co species, and (e,f) $RhCrO_x/SrTiO_3$:Al coloaded with 0.3 wt% Co species and modified with 0.3 wt% TiO_2 (a,c,e) before and (b,d,f) after water splitting reaction over one week time span under irradiation by high-pressure Hg lamp (λ > 300 nm). The data in (a) were acquired following a 1 h reaction and after the sample was collected to remove water-soluble Cr^{6+} species generated by calcination in air at 623 K.

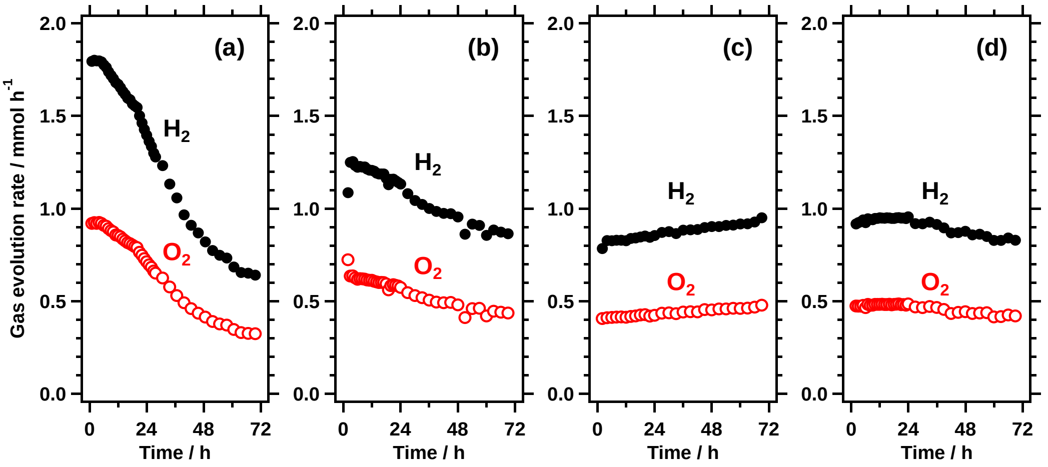

Figure S4. Time courses of water splitting activity of $CoOOH/RhCrO_x/SrTiO_3$:Al modified with TiO_2 by *in situ* photodeposition at TiO_2 amounts of (a) 0, (b) 0.1, (c) 0.3 and (d) 0.5 wt%. Reaction conditions: photocatalyst, 0.1 g; reaction solution, 100 mL distilled water; reaction temperature, 291 K; light source, 300 W Xe lamp (300 nm < λ < 500 nm).

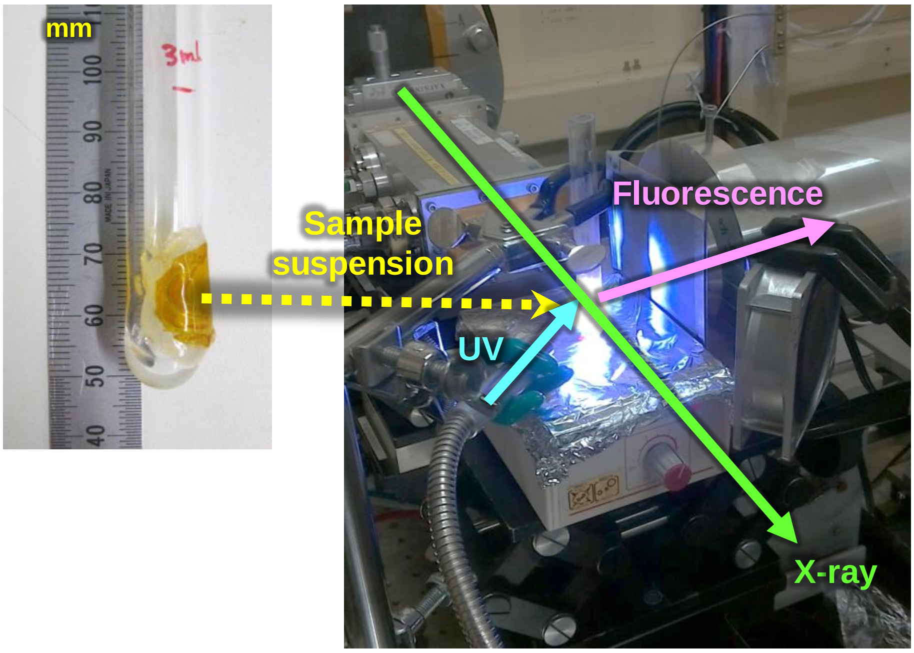

Figure S5. Experimental setup for operando XAFS spectroscopy of $CoOOH/RhCrO_x/SrTiO_3$:Al photocatalyst suspension.
